# Simplified detection of the hybridized DNA using a graphene field effect transistor

**DOI:** 10.1080/14686996.2016.1253408

**Published:** 2017-01-10

**Authors:** Arun Kumar Manoharan, Shanmugavel Chinnathambi, Ramasamy Jayavel, Nobutaka Hanagata

**Affiliations:** ^a^ Centre for Nanoscience and Technology, Anna University, Chennai, India; ^b^ Nanotechnology Innovation Station, National Institute for Materials Science, Tsukuba, Japan; ^c^ Graduate School of Life Science, Hokkaido University, Sapporo, Japan

**Keywords:** Graphene, field effect transistor, Dirac point, DNA, 40 Optical, magnetic and electronic device materials, 208 Sensors and actuators

## Abstract

Detection of disease-related gene expression by DNA hybridization is a useful diagnostic method. In this study a monolayer graphene field effect transistor (GFET) was fabricated for the detection of a particular single-stranded DNA (target DNA). The probe DNA, which is a single-stranded DNA with a complementary nucleotide sequence, was directly immobilized onto the graphene surface without any linker. The V_Dirac_ was shifted to the negative direction in the probe DNA immobilization. A further shift of V_Dirac_ in the negative direction was observed when the target DNA was applied to GFET, but no shift was observed upon the application of non-complementary mismatched DNA. Direct immobilization of double-stranded DNA onto the graphene surface also shifted the V_Dirac_ in the negative direction to the same extent as that of the shift induced by the immobilization of probe DNA and following target DNA application. These results suggest that the further shift of V_Dirac_ after application of the target DNA to the GFET was caused by the hybridization between the probe DNA and target DNA.

## Graphical abstract

**Figure:**
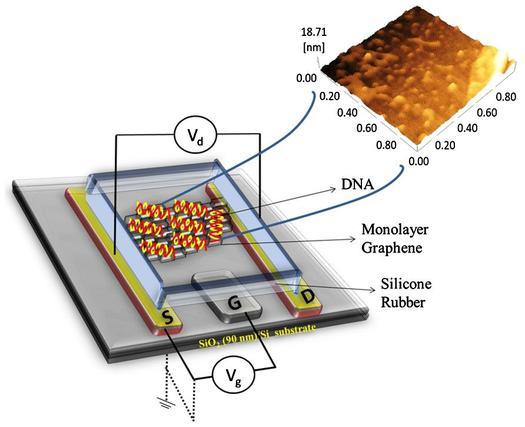

## 1. Introduction

Recently, graphene, a single atom thick and two-dimensional carbon material has been attracting much attention for use in chemical and biological sensors.[1–7] The interaction between nanomaterials and macromolecules in sensing technologies has gained much importance and provides a versatile platform.[8] Moreover, carbon nanotubes,[9,10] graphene oxide [2,3] (GO) and reduced graphene oxide [6] (rGO) have been widely referred for the detection of chemicals,[4,5] proteins,[11] DNA hybridization,[12,13] and cellular activities.[14] Graphene has attracted attention because of its high charge sensitivity to environmental disruptions, and hence has been used in sensors due to its electrical and optical properties. Graphene based transistors [12,15–18] were developed as simple and convenient biological sensors. In particular, the graphene field effect transistor [19–21] (GFET) for DNA (deoxyribonucleic acid) sensors has been developed by several research groups.[13,22–25] Recent achievements in the production of monolayer graphene by chemical vapor deposition [1,26–29] (CVD) and mechanical exfoliation [13,18,27] provide a new platform for developing biological sensors. Detection of electrical conduction was similar for all graphene sheets, with the transfer characteristics for graphene oxide and reduced graphene oxide exhibiting a reduced current ratio when compared with that of monolayer graphene prepared by the CVD and exfoliation method. Hence, graphene has high conductivity and high carrier mobility, and thus it can sense DNA molecules or ions in buffer solutions. While measuring electrical detection of DNA molecules, variations occur specifically by changes in conductivity. Charge conductivity easily changes the device performance. Changes in conductivity may be affected by defects in the rGO, which influences detection performance. The interaction between rGO and DNA can also be affected by the defects present in rGO sheets. Device performance reported by Lin et al. [22] revealed a shift of the transfer curve (V_Dirac_ point) to the left (negative), clearly describing the n-doping of nucleobase DNA on the graphene and sometimes revealed shifts to the right (positive), referring to p-doping (hole carrier concentration).

These devices create a new opening for the development of advanced biosensors. Detection of disease-related genes that are not expressed in normal individuals is one of the useful methods for diagnosis. DNA hybridization, the formation of double-stranded DNA by hydrogen bonds between nucleotide bases of single-stranded DNA and its complementary DNA, is used to detect specific DNA expression. In a DNA sensing experiment, phosphate groups play a vital role, which creates van der Waals dispersion forces [30–32] and electronic interactions such as induced dipole [33] and doping [22] between the DNA nucleobases and graphene. Optical detection is generally used for detecting DNA hybridization. In this method, the target DNA is labeled with a fluorescence dye, and the fluorescence intensity is measured from the target DNA hybridized to the probe DNA immobilized on the substrate. However, this procedure requires an expensive laser scanner for the detection of fluorescence intensity and a complicated procedure for the labeling process, which limits the use of this method on medical sites.[13] Among these approaches, label-free electrical detection has been studied extensively, as it does not require fluorescent tags.[22,23] Thus, the GFET has been developed as a label-free sensing device for DNA detection. Detection of label-free target DNA using GFET is based on the change in Fermi level [18,34,35] upon hybridization of the target DNA with the probe DNA on graphene. In most GFETs, probe DNA is immobilized on graphene through linkers such as pyrene derivatives,[13] gold nanoparticles,[23] and biothylated BSA.[12]

In this study, we have fabricated a GFET based on the mechanical exfoliation method with an inbuilt reference electrode acting as an electrolyte gate electrode. We observed the direct adsorption of single- and double-stranded DNA to carbon nanohorns, graphene sheets, and graphene oxide sheets in the solution. These observations suggest that probe DNA can be directly immobilized onto the graphene on substrates without linker modifications. However, we could not immobilize the probe DNA in the solution directly onto the graphene surface formed on the substrate. These observations reveal that only the DNA suspended in the solution has the potential to be adsorbed onto the carbon materials. Thus, we evaporated water from the probe DNA solution dropped onto the graphene surface and desiccated the probe DNA. Atomic force microscopy revealed that the probe DNA was adsorbed onto the graphene surface, even after washing the desiccated surface. Herein, the desiccation process is essential for the direct immobilization of probe DNA on the graphene surface. The target DNA solution was applied to the probe DNA solution because the target DNA solution could not be directly adsorbed onto the graphene surface. This paper describes the detection of target DNA hybridized with the probe DNA directly immobilized on the graphene surface in a GFET. In recent cases of cancer therapy, such as DNA based therapies, messenger RNA (mRNA) are also used as alternatives. mRNA has a good capability to classify cancer subtypes. This device can support mRNA detection and provides the best platform for cancer detection.

## 2. Materials and methods

### 2.1. Fabrication of GFET

The monolayer graphene was deposited onto a Si substrate covered with a 90-nm-thick SiO_2_ layer by the mechanical exfoliation of kish graphite (Covalent Material Co., Ltd, Tokyo, Japan) with an adhesive tape. The monolayer graphene on the substrate was identified by optical microscopy (S8 APO+EC3, Leica Microsystems, Wetzlar, Germany). Reflection of light in the optical microscope can easily identify the number of layers. The number of layers present on the substrate was confirmed by Raman spectroscopy (532 nm excitation, RAMANplus, Nanophoton, Osaka, Japan). The electrodes and contact pads for the metal contact on the corresponding monolayer graphene were designed using CAD Software (Vectorworks 2012 tool, A&A Co. Ltd, Tokyo, Japan). The substrate was first coated with a hydrophobic hexamethyldisilazane (HMDS, Merck Performance Materials, Darmstadt, Germany) layer, and LOR5A (Microchem Corp., Westborough, MA, USA) was then deposited on the substrate, followed by baking at 180 °C for 5 min. This acted as a protective layer, and AZ5214E (Merck Performance Materials) was then deposited on the substrate followed by baking at 110 °C for 2 min, to act as a photoresistant layer. The etching pattern of the substrate was developed by scanning maskless lithography (DL-1000/NC2P, NanoSystem Solutions Inc., Okinawa, Japan), with a semiconductor laser with a power of 1 W cm^–2^ at 405 nm, exposure to a dose of 85 mJ cm^–2^ in order to pattern the AZ-resist. The resist was developed by a standard developer (2.38% solution of tetramethylammonium hydroxide, TMAH, MicroChem Corp.) at 2 min and 30 s followed by rinsing the substrate in deionized (DI) water for 30 s. The electrodes (Ti/Au~ 10/200 nm) were prepared by the evaporation of metal using an electron-gun evaporator (RDEB-1206 K, RDEC Inc., Ibaraki, Japan). The lift-off method was adopted to remove the metal deposition from the unwanted regions by using N-methyl-2-pyrrolidone (NMP, MicroChem Corp.) at 80 °C for 1 h and 15 min, followed by washing the substrate with acetone and isopropanol. The electrodes were passivated by an insulator layer (Al_2_O_3_) using atomic layer deposition (ALD) at 0.8 Å/cycle. HMDS and AZ5214E were again deposited on the substrate and baked at 110 °C for 2 min, followed by etching the pattern using scanning maskless lithography. The resist was prepared by the developer (2.38% TMAH solution) for 90 s, then rinsed in DI water for 30 s and baked at 110 °C for 5 min. Al_2_O_3_ was etched from the pattern by keeping the substrate in the etchant for 10 min and baking at 110 °C for 5 min, twice successfully. Here, the developer and etchant were the same solution (2.38% TMAH solution). The lift-off process was conducted for 45 min to remove the Al_2_O_3_ from the unwanted regions. The source and drain (Ti/Au~10/200 nm) were made successfully. Similar fabrication steps were followed for making the gate (Ti/Pt~150/50 nm). The electrodes were fabricated on the substrate to form the reservoir, which acts as an insulator. The reservoir was formed by using silicone rubber (TSE382-C, Tanac Co., Ltd, Gifu, Japan) for particular regions. The insulating layer was used to obviate the leakage current from the metal contacts, and the electrodes were protected from electrolytes using rubber for insulation.

### 2.2. Detection of target DNA hybridization with probe DNA

Single-stranded probe DNA (24 base-5′ TCG-TCG-TTT-TGT-CGT-TTT-GTC-GTT 3′) and single-stranded target DNA (24 base-3′ AGC-AGC-AAA-ACA-GCA-AAA-CAG-CAA 5′) were purchased from Greiner Bio-One, (Kremsmunster, Austria). The graphene device was incubated in phosphate-buffered saline (PBS, pH 7.4) for 2 h, the probe DNA (10 µM) in PBS was dropped onto the graphene surface and incubated at room temperature. During the incubation, water was completely evaporated, and the probe DNA was desiccated. The desiccated probe DNA was washed twice with DI water to remove the unbound probe DNA. The target DNA in PBS was then added to the probe DNA and incubated at room temperature for 4 h for hybridization. Non-complementary DNA was also applied to the probe DNA as a control. Electrical signals of graphene FETs were measured with a semiconductor parameter analyzer (4200-SCS, Keithley, Cleveland, OH, USA) equipped with a liquid-nitrogen probe (SB-LN2 ps, System Brain, Tokyo, Japan). The Ti/Au electrodes act as a source and drain, and the Ti/Pt electrode acted as a gate. The electrical properties were studied for both the back gate and top gate of the device. During measurement, the back gate connection was made at the SiO_2_/Si substrate, and the top gate connection was made at the Ti/Pt electrode. The transfer characteristics were measured using both back gate and top gate. DNA hybridization was measured once the device was thoroughly checked by the analyzer.

### 2.3. Surface topography of PBS/single-stranded probe DNA/double-stranded DNA on graphene

Tapping mode atomic force microscopy (AFM, Olympus – OLS3500, Tokyo, Japan) technique was used to study the surface topography of PBS, single-stranded probe DNA, and double-stranded DNA on graphene.

## 3. Results and discussion

### 3.1. Identification of graphene layers and fabrication of GFET

**Figure 1.**
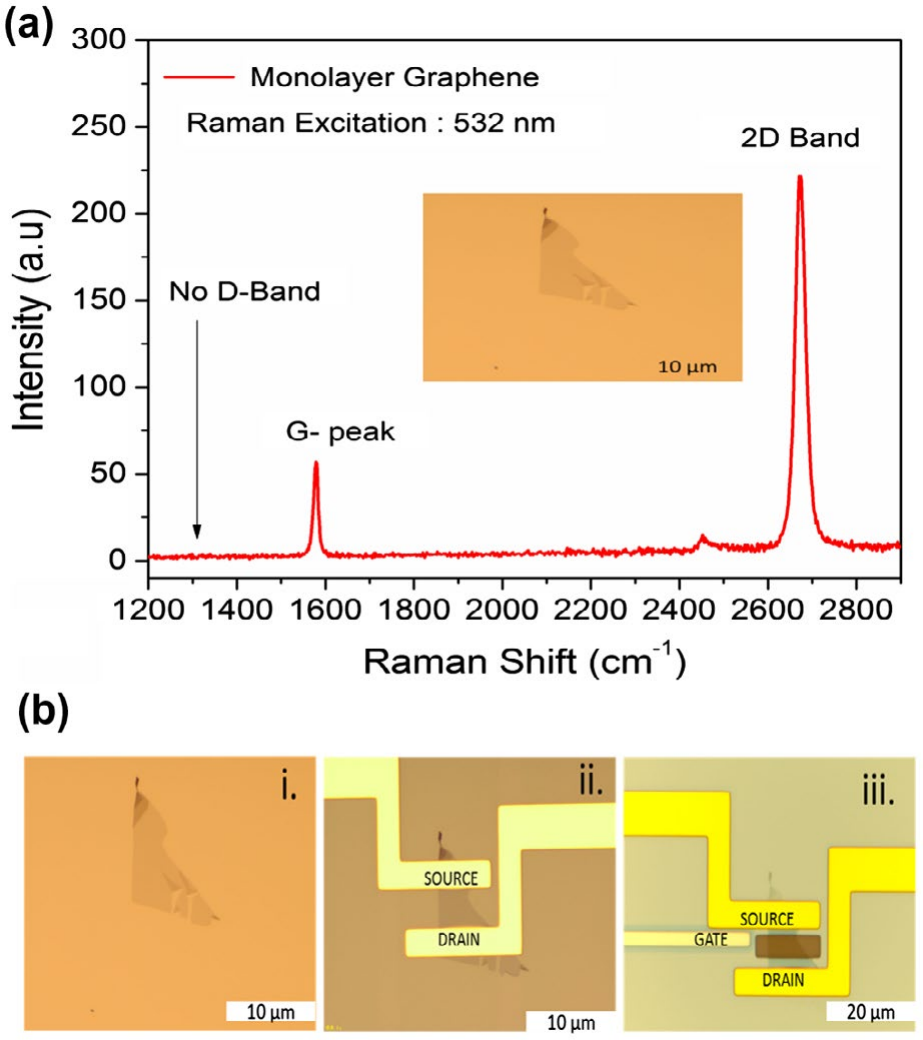
(a) Raman spectrum of monolayer graphene, which was shifted onto the SiO_2_/Si substrate using scotch tape. A strong peak was observed at 2672 ${\text{cm}}^{-1}$. (b) Optical image of monolayer graphene, which illustrates the different stages of GFET device fabrication. (i) Monolayer graphene, (ii) fabrication of source-drain electrodes (Ti/Au), and (iii) fabrication of top gate electrode (Ti/Pt).

**Figure 2.**
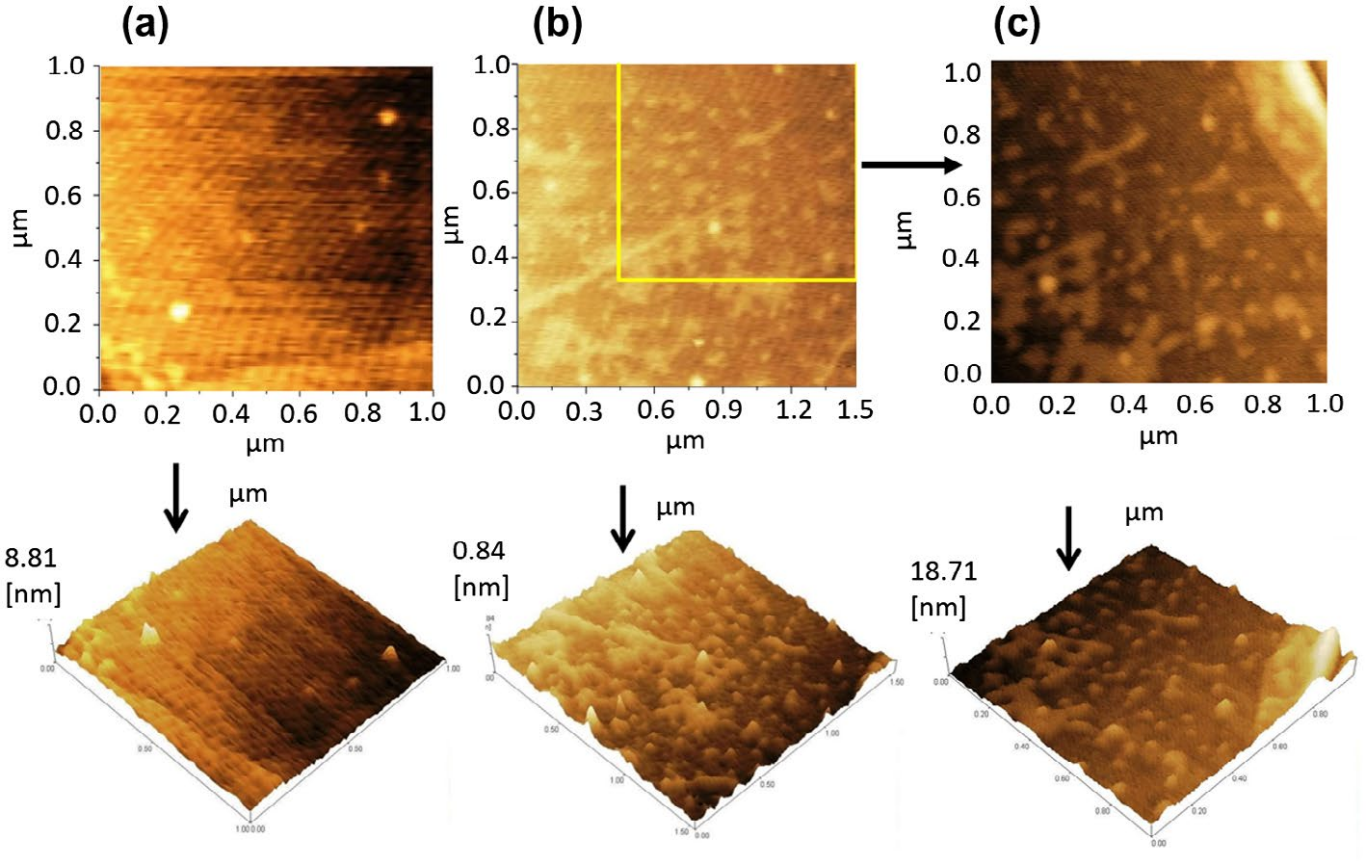
Tapping mode AFM images of graphene. Analyses were based on PBS and single-stranded probe DNA. (a) 2D and 3D AFM images of graphene with PBS solution, (scan area: 1 µm × 1 µm). (b) 2D and 3D AFM images of graphene with single-stranded probe DNA, (scan area: 1.5 µm × 1.5 µm). The colored line illustrates the magnified area of the image (c). (c) 2D and 3D AFM images (magnified image of (b)) of graphene with single-stranded probe DNA, (scan area: 1 µm × 1 µm).

**Figure 3.**
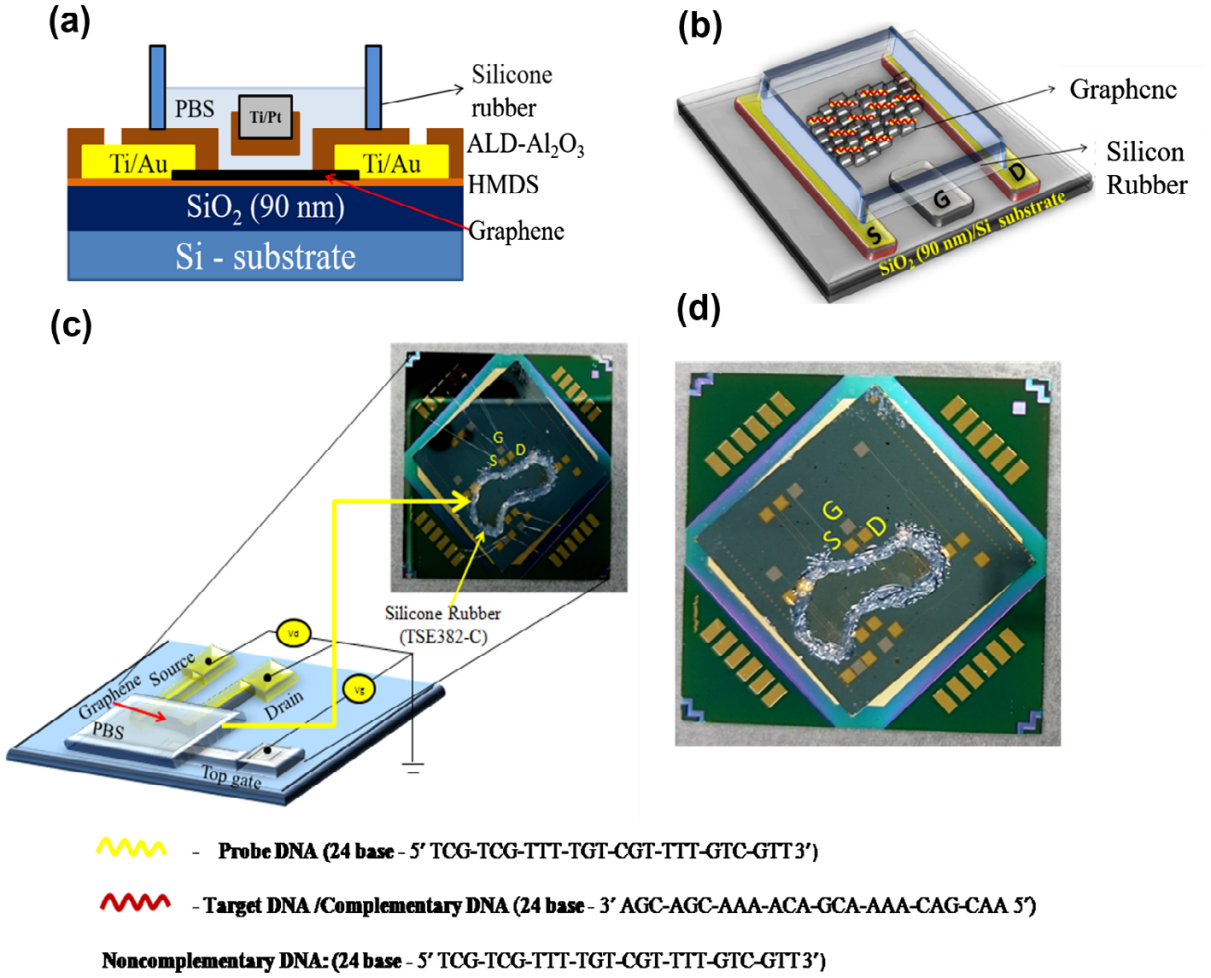
A schematic diagram of the graphene field effect transistor (GFET) with an inbuilt gate for electrolyte measurements. Ti and Au were used as the source (S) and drain (D) electrodes, respectively, and another material (Ti/Pt) was used as the inbuilt gate (G) electrode for GFET measurements. (a) Cross-sectional view of the graphene site. (b) Top view of the GFET device, the colored line indicates the DNA sequence used for the experiments. (c) Experimental setup of the GFET (color line). The image shows the entire device placed on the circuit board for GFET measurements. (d) Photograph of the GFET device, which contains the source (S), drain (D), and the inbuilt gate (G) electrodes.

Graphene was deposited on a SiO_2_/Si substrate by the exfoliation method, and characterized by optical microscopy and Raman spectroscopy as shown in Figure 1(a). Its monolayer structure was confirmed from the position, width and intensity ratio of the G and 2D peaks observed at 1580 and 2672 ${\text{cm}}^{-1}$, respectively. The defect-induced D-band at ~1350 ${\text{cm}}^{-1}$ was not detected, indicating the high structural quality of the sample. The absence of D-band confirmed that there are no defects, thus confirming the high quality of monolayer graphene used in this study. After confirming monolayer graphene on the substrate, the fabrication process was performed. The optical image of monolayer graphene is shown in Figure 1(b, i). The optical image of the source and the drain electrodes (Ti/Au) on the graphene is shown in Figure 1(b, ii) and the inbuilt gate electrode (Ti/Pt) on the substrate in Figure 1(b, iii). Surface topography was observed for PBS, single-stranded DNA, and double-stranded DNA on the graphene surface using AFM. Figure 2(a) shows the AFM image of PBS, and Figure 2(b) and (c) illustrates the AFM image of the single-stranded probe DNA. The AFM images clearly demonstrate the immobilization of single-stranded probe DNA on the graphene surface. AFM images for the immobilization of double-stranded DNA on the graphene surface are shown in Figure S3 (see supplementary information). The complete GFET transistor with the inbuilt gate was fabricated for electrolyte measurements, and its cross sectional view and the top view of the graphene device are shown in Figure 3(a) and (b). The experimental setup for the device using reservoir is shown in Figure 3(c) and (d). The transfer characteristics observed for the DNA treatments are explained in the following section.

### 3.2. Output characteristics of back-gated GFET

**Figure 4.**
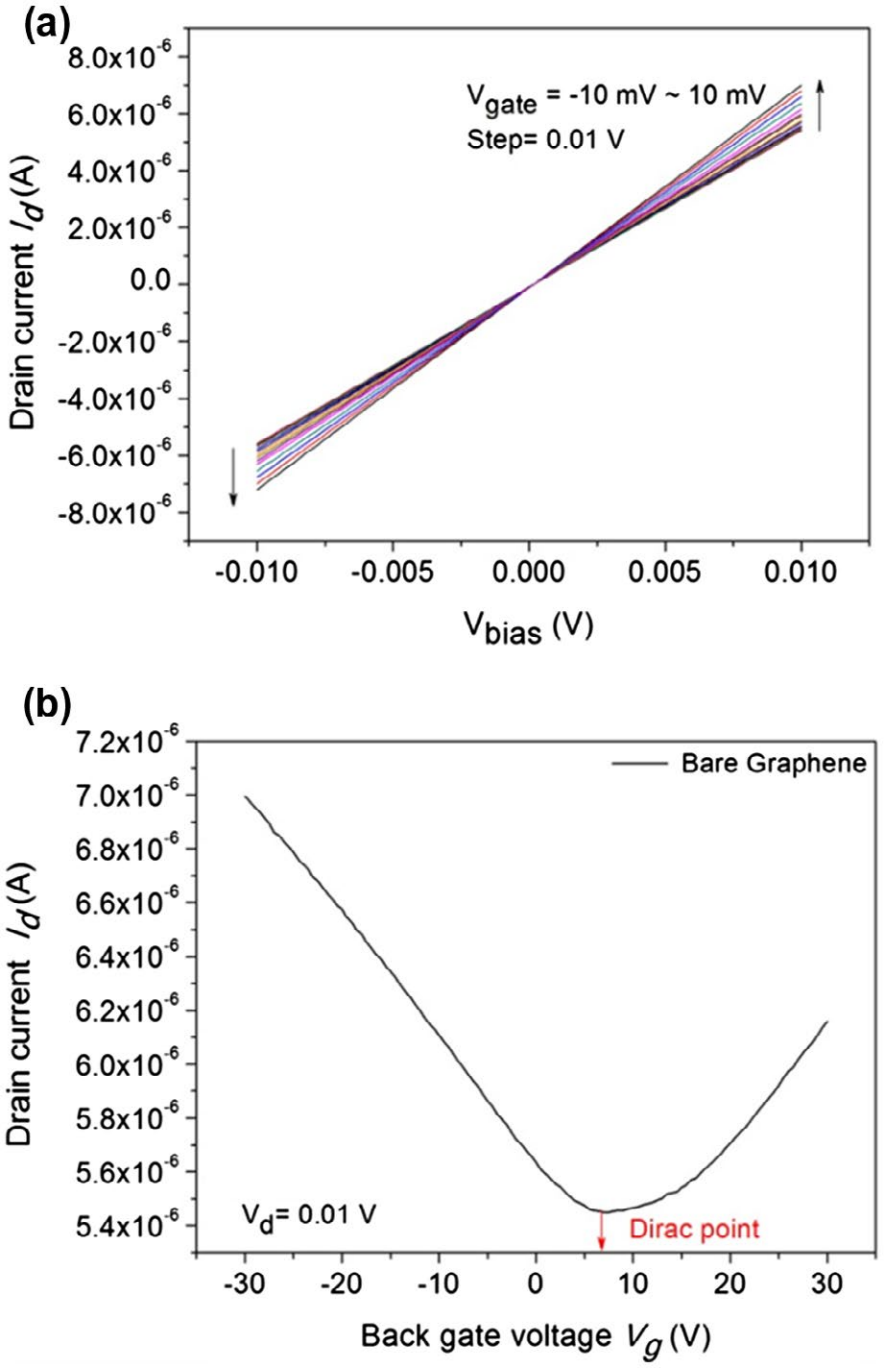
Output and transfer characteristics of the back-gated graphene device. (a) Output characteristics with drain current vs*.* bias voltage measured at various V_g_ ranging from –10 MV to 10 MV with a step of 0.01 V. (b) The transfer curve for bare graphene between the drain current *vs.* back gate voltage at V_d_ = 0.01 V. The colored line shows the Dirac point of the device at V_g_ = 0.6 V.

The output characteristics of the graphene device were measured using the Keithley parametric analyzer. Figure 4(a) shows the drain current *vs.* bias voltage characteristics of the GFET device (referred to as device #1) at various gate voltages. The gate voltage was varied from V_g_ = −10 to 10 mV at 0.01 V steps. The contacts made on each device were characterized by measuring the output characteristics (I_d_ Vs. V_bias_). The drain current of the device linearly increases with the bias voltage, illustrating a linear behavior for both the Ti/Au contacts in the device at low bias. It can be observed that all lines cross the zero point at the zero bias for all back gate voltages. The output characteristics of GFET confirmed the strong binding between the graphene and the metal contacts.

### 3.3. Transfer characteristics of back-gated GFET

Figure 4(b) shows the transfer characteristics of the GFET device (device #1) between the drain current and the back gate voltage at different drain voltages (at V_d_ = 0.01 V). The back gate voltage (V_g_) was set in the range –35 to 35 V. The contacts made on each device were characterized by measuring the transfer characteristics (I_d_ Vs. V_bg_). The back gate voltage was applied to the SiO_2_/Si substrate, while the source pad was grounded and the drain pad biased at 10 mV. The graph shows that the I_d_ of bare graphene was decreased once and then increased, indicating the typical transfer characteristics of the graphene FET, which were also reported by Ohno et al. [13]. Here, monolayer graphene acts as a channel between the source and the drain. The carrier density and the type of carrier (electrons/holes) in the channel are presided over by the potential differences between the channel and the gate. The positive gate voltages endorse the electron concentration in the n-type channel, whereas the negative gate voltages support the hole concentration in the p-type channel. The hole and electron concentration activity gives rise to the transfer characteristics, and it is separated by the Dirac point. Single layer graphene has a zero band gap semimetal; thus, Dirac fermion can be converted from electron to hole (or from hole to electron) continuously under the electric field as reported by Zhan et al*.* [1]. The charge carrier conversion can change incessantly by increasing the ambipolar gate voltage.

### 3.4. Transfer characteristics of solution-gated GFET

**Figure 5.**
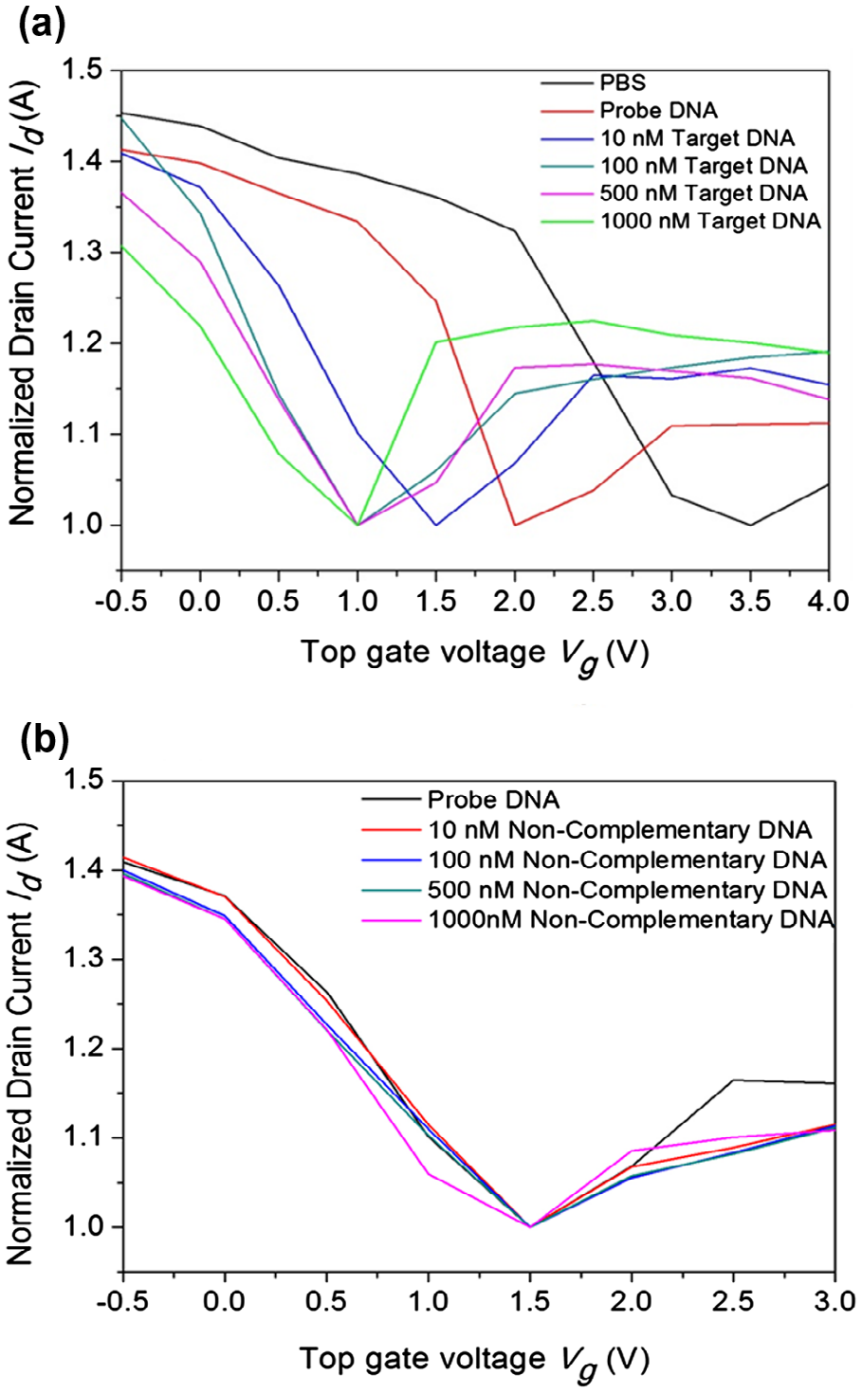
Transfer characteristics of the top-gated graphene device (device #1). The transfer curve of the graphene device measured between the drain current vs. top gate voltage at V_d_ = 0.01 V. In device #1: (a) The measurements were performed for PBS (black line), after adding the probe DNA (dark red line, 10 µM), and hybridization with target DNA at concentrations ranging from 10 to 1000 nM. (b) Hybridization with non-complementary DNA with concentrations ranging from 10 to 1000 nM.

The transfer characteristics of the GFET between the drain current and top gate voltage at the drain voltage V_d_ = 0.01 V is shown in Figure 5. The top gate was fabricated along the insulator (such as Al_2_O_3_), which acts as a gate dielectric on the graphene substrate. The source pad was grounded, and the drain pad was biased at 10 mV. In this fabrication, the monolayer graphene was carefully chosen and covered with photoresist excluding the graphene region (Figure 1(b, iii)). The solutions (PBS, DNA) were added drop wise. Here, it should be noted that the contact area between the Pt/Ti electrode and the DNA solution is much larger than the contact area between the DNA solution and graphene. Moreover, the size of the Pt/Ti electrode is also much larger than the size of the graphene layer. The accurate bias in this analysis may be defined as the potential difference from the reference electrode using a three terminal setup as reported by Uesugi et al. [36]. The potential drop at the Pt/Ti electrode is expected to be very small, and therefore it is not considered. The solution-gated GFET have recently been used for the detection of DNA hybridization with high sensitivity. Compared to back-gated GFETs, top-gated GFETs show more flexibility in circuit application, as reported by Zhan et al. [1]. The carrier mobility in the top-gated device was much lower than that in the back-gated device. However, the back gated GFET was not suitable for the analyte solution considered in DNA sensors. In the analyte solution, leakage current may occur between the channel and gate, which would be a critical factor for device reliability. To avoid this critical factor and to improve device reliability, the top-gated GFETs were fabricated with dielectrics.

However, the transfer characteristics of the GFET device (Figure 4(b)) were measured directly with bare graphene. It was observed that the V-I curve was initially decreased and then increased in a trend linear manner (smooth curve), due to the higher electron conductivity of graphene. In the case of the transfer characteristics measured in the presence of desiccated PBS and DNA (Figure 5), the V-I curve are in a jagged manner. The origin of this jagged manner is due to differences in electron conductivity between graphene and DNA. Usually, DNA molecules on the graphene surface act as the charge impurities and affect the electron conductivity of graphene. The doping effect on the graphene films is discussed in detail in the following sections.

The band gap tunability plays a beneficial role for the semi-conductive property of graphene, and the current-voltage characteristics clearly explain the ambipolarity of graphene. The fabrication steps are clearly shown in Figure 1(b). The fabrication and characterization of each device were clearly checked before the hybridization process. The transfer characteristics of the device (V_d_ = 0.01 V) were measured with PBS, probe DNA, and hybridization of different target DNA concentrations as shown in Figure 5(a). Initially, the characteristics of the pure PBS solution were studied, and the ambipolar behavior of the device was observed based on the hole and electron transport for the consequent gate voltage, V_Dirac_ value achieved as positive (V = 3.5 V). After measuring the PBS buffer solution, the probe DNA (10 µM) was dropped into the reservoir and desiccated, by which the graphene surface was interacted with nucleotides by the van der Waals dispersion forces. Interestingly, the V_Dirac_ point was shifted to the left (i.e. Dirac point is shifted from 3.5 V to 2.0 V) because of the n-doping of DNA on graphene. Further, the target DNA was dropped on to the reservoir and allowed to hybridize. Electronic measurements were performed after removing the unbound DNA from the reservoir with DI water. The experiment was initiated with 10 nM of target DNA, and the Dirac point was again shifted towards the left after the DNA hybridization. This reveals that the complementary target DNAs interacted with the probe DNA on the graphene surface, resulting in an n-doping effect. With further increase in target DNA concentration (100, 500, and 1000 nM), the shift of the Dirac point also increased towards the left because of enhancement of the n-doping effect. However, this shift was not observed at higher concentrations of the target DNA owing to the limitation of probe DNA. On the other hand, the Dirac shift did not occur for the measurements carried out using non-complementary DNA, possibly because of the decreased n-doping effect as shown in Figure 5(b). Therefore, these charge transfer characteristics demonstrate that the GFET device can effectively identify DNA hybridization.

In this system, the 24-mer probe and the target DNA (approximately 8 nm in length) [37] were added to the graphene surface, which is influenced by interaction from the nucleosides and the phosphate groups. Herein, the DNA is decorated on the graphene without any kind of linker. Thus, the Debye length is less than 1 nm,[22] and most of the negative charges should lie outside the Debye length. Prospectively, the nucleosides attached to graphene induce the Dirac point to shift towards the left side. Once the target DNA is added, the hybridized DNA becomes stiff with outward projection from the graphene surface. Therefore, the hole carrier concentration is controlled by the negative charges adjacent to the graphene surface even though the probe DNA strongly adheres to the graphene surfaces as illustrated by the left-shift of the V_Dirac_ point (Figure 5). The hole carrier concentration is dominated as mentioned above, and the negative phosphate ions of the hybridized DNA segments can still contribute to the overall hole carrier concentration.

In order to evaluate the working strategy of the graphene device, electrical measurements were repeated for other devices (device #2 and #3, see supplementary information). In device #2, the probe DNA (10 µM) was desiccated; herein, the target DNA (10 µM) was dropped and allowed to hybridize. The result shows that addition of target DNA helps to shift the V_Dirac_ point towards the left (from 0.01 V to –0.2 V), as shown in Figure S2. In device #3, the double-stranded DNA (dsDNA, 10 µM) was dropped and desiccated shifting the Dirac point towards the left (from 0.6 V to –1 V) as shown in Figure S5. Hence, the experimental results of device #1, device #2, and device #3 clearly indicate that the negative shift of the Dirac point in the transfer curves before and after the addition of DNA clearly reveal the electron doping from the negative charges. The negative shift in the threshold voltage after immobilization of probe DNA has been attributed to electron transfer from the electron-rich aromatic nucleotide bases in DNA and to the transfer of electrons from DNA to graphene. In the n-doping model, the possibility of electron transfer is because of the higher electron affinity of graphene. To confirm the interaction between DNA and graphene surfaces in the present study, the process was repeated for different concentrations of DNA, and the negative shift observed in the transfer characteristics (Figure 5) was confirmed to be due to electron transfer from DNA to graphene. In comparison, the transfer characteristics of these devices indicate that the Dirac point shift varies (either increases or decreases) with varying DNA concentrations. The hole carrier concentration of graphene was attained only for complementary DNA (left shift) and was absent for non-complementary DNA. The hole carrier concentration of graphene increases with the addition of dsDNA because of its fully hybridized nature. After summarizing all the transfer characteristics of the graphene devices (devices #1, #2, and #3), the hole carrier concentration of graphene was governed by negatively charged ions (phosphate group) of DNA. The interaction between the nucleotides and graphene clearly explored the n-doping electronic interaction during the shifting of the Dirac point. Based on these results, it can be observed that graphene surfaces play a major role in the shifting of V_Dirac_, which provides a new path to stimulate further research on graphene based biosensors.

## 4. Conclusions

GFET was fabricated based on the monolayer graphene with an inbuilt reference electrode, which acts as an electrolyte gate electrode for detecting DNA hybridization. The linearity between metallic contacts and graphene devices was clearly monitored from the output characteristics and the back gate performance of all devices and then used for the detection of gene expression by DNA hybridization. The V_Dirac_ or carrier concentration of the device was determined with different treatments such as ssDNA, dsDNA, and DNA hybridization. Interestingly, the shifting of the Dirac point for ssDNA and complementary DNA towards the left side indicates an n-doping effect, whereas a shift with non-complementary DNA was not observed. The variation in the transfer characteristics between the probe DNA with the target DNA, and the probe DNA with the non-complementary DNA, reveals that the newly fabricated GFET is more sensitive for the specific detection of DNA nucleotides. Further development of this GFET device could be explored with enhanced performance for future biosensor applications, particularly in the detection of genetic diseases.

## Disclosure statement

No potential conflict of interest was reported by the authors.

## Funding

This work was supported by NIMS Molecule & Material Synthesis Platform in ‘Nanotechnology Platform Project’ operated by the Ministry of Education, Culture, Sports, Science, and Technology (MEXT), Japan.

## Supplementary Material

The underlying research materials for this article can be accessed at http://dx.doi.org/10.1080/14686996.2016.1253408

## Supplementary Material

TSTA_A_1253408_Supplementary_Information.pdf
