# Supplementary material for: Simplified detection of the hybridized DNA using a graphene field effect transistor
Source: Sci Technol Adv Mater. 2017 Jan 10;18(1):43–50. doi: 10.1080/14686996.2016.1253408 (PMC5256270; doi:10.1080/14686996.2016.1253408)
Supplement: TSTA_A_1253408_Supplementary_Information.pdf [file tsta_a_1253408_sm4488.pdf]

Supplementary Information

Simplified detection of the hybridized DNA using a graphene field effect transistor

Arun Kumar Manoharan[a,b], Shanmugavel Chinnathambi[b,c], Ramasamy Jayavel[a] and Nobutaka Hanagata[b,d]

[a]Centre for Nanoscience and Technology, Anna University, Chennai, India;

[b]Nanotechnology Innovation Station, National Institute for Materials Science, Tsukuba, Japan;

[c]JSPS Research Fellow, Tokyo, Japan;

[d]Graduate School of Life Science, Hokkaido University, Sapporo, Japan

Device fabrication and characteristics for additional graphene field effect transistors (GFETs – device #2 and device #3):

Device #2:

We fabricated another device (GFET – device #2) to evaluate the working strategy of graphene devices. After checking the fabrication process and the device characteristics, we used the device for DNA hybridization. The output characteristics and the transfer characteristics of device #2 were measured for the drain voltage V_d = 0.01 V with the gate voltage varying from V_g= -10 to 10 mV as shown in figure S1. Similar to device #1, the output characteristics of device #2 also have linearity and good metal contact. The transfer characteristics of device #2 were comparatively similar to those of device #1. As per the experimental procedure, the single-stranded probe DNA (10 µM) was desiccated onto the device, and the target DNA (10µM) was dropped and allowed to hybridize. The electrical measurement was performed in each treatment. The Dirac point for the PBS was achieved at V_g = 0.1 V as shown in figure S2. After the desiccation of probe DNA, the Dirac point was shifted to the left side at V_g = 0.01 V and for the target DNA, it was again shifted towards the left at V_g = - 0.2 V. In device #2 (figure S2), the shifting of the Dirac point towards the left is comparatively higher than that for device #1 (as discussed in this paper – section 3.4). This could be due to the higher concentration of target DNA (i.e. 10 µM). Both the transfer characteristics (device #1 and device #2) demonstrated that the large shift observed at a higher concentration of target DNA is due to the enhancement of DNA hybridization.

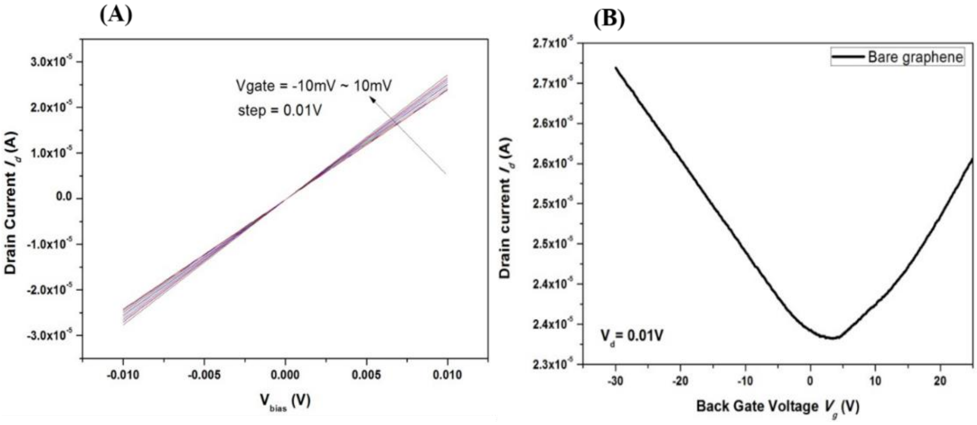

Figure S1. Output and transfer characteristics of device #2. (a) Drain current *vs.* Bias voltage measured at various V_{gate} (from -10 mV to 10 mV) with a step of 0.01V. (b) The transfer curve for bare graphene between the drain current *vs.* back gate voltage at V_d = 0.01 V.

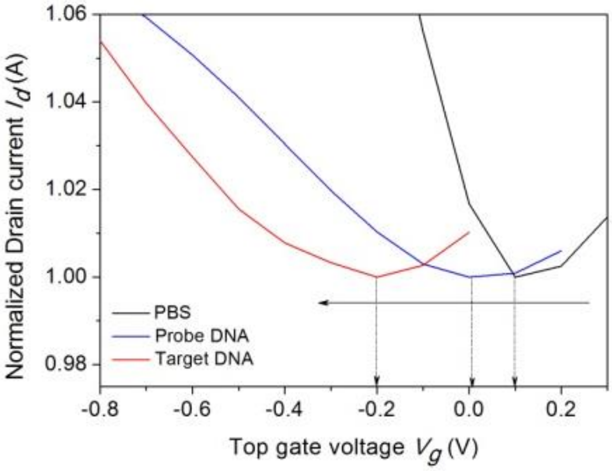

Figure S2. Transfer characteristics of the device for the observation of PBS (black line), probe DNA (10 μM, blue line), and the target DNA (10 μM, red line). The arrow shows the shifting of Dirac point for each treatment.

Device # 3:

In addition, device #3 was fabricated to study the transfer characteristics of dsDNA (10μM). Prior to the analysis, the surface topography of the desiccated dsDNA on the graphene surface was observed using tapping mode AFM as shown in figure S3. The output and transfer characteristics of the device are shown in figure S4. Figure S5 shows the transfer characteristics of desiccated dsDNA, in which the Dirac point was achieved for PBS at $V_g = 0.6$ V and for dsDNA at $V_g = -1$ V. The arrow clearly indicates Dirac point shifting. In general, dsDNA is fully hybridized, thus the shifting of Dirac point was comprehensively higher than that observed for ssDNA.

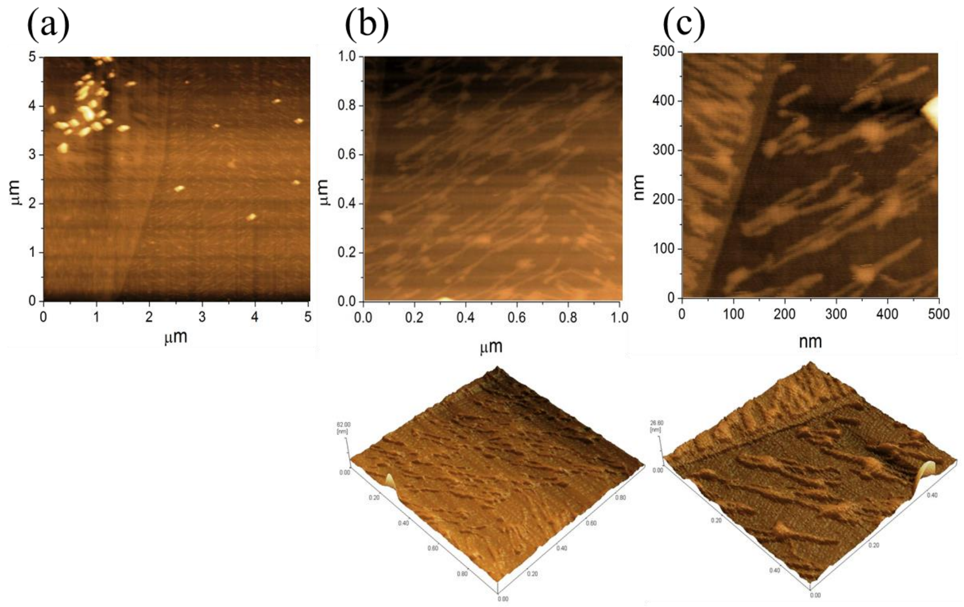

Figure S3. Tapping mode AFM images of graphene, based on double-stranded DNA (dsDNA). (a) 2D AFM images of graphene with dsDNA (scan area: 5 μm × 5 μm). (b) 2D and 3D AFM images of graphene with dsDNA, (scan area: 1 μm × 1 μm). (c) 2D and 3D AFM images of graphene with dsDNA, (scan area: 500 nm × 500 nm).

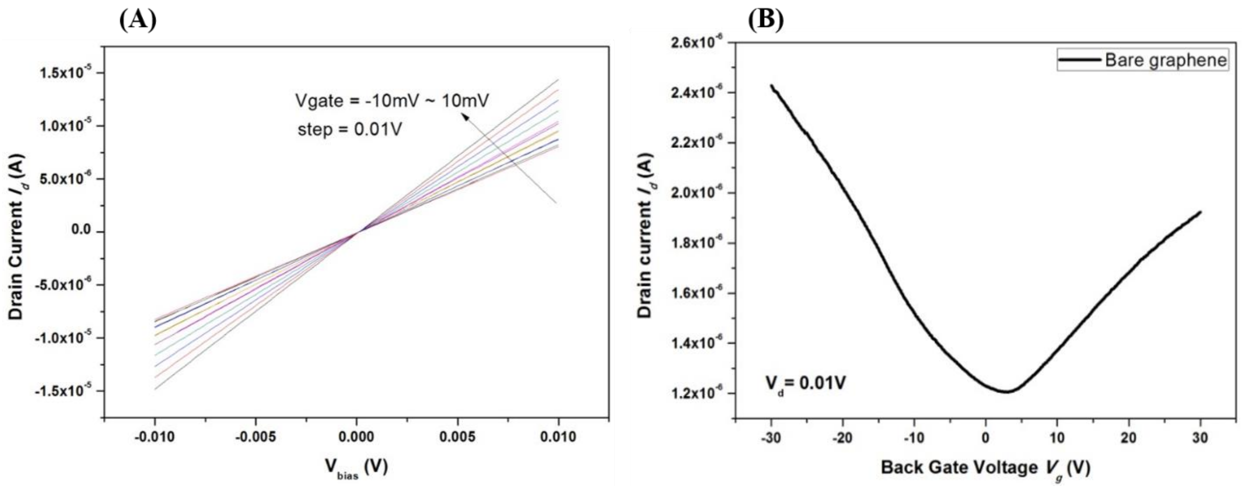

Figure S4. Output and transfer characteristics of device #3. (a) Drain current *vs.* bias voltage measured at various V_{gate} (from -10 mV to 10 mV) with a step of 0.01 V. (b) The transfer curve for bare graphene between the drain current *vs.* back gate voltage at V_d = 0.01 V.

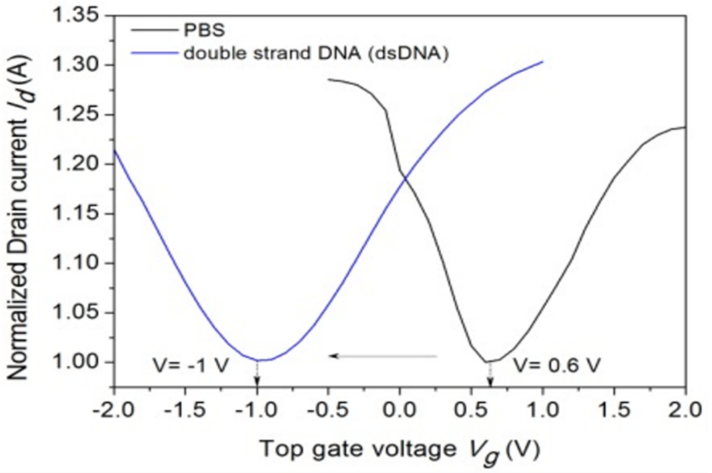

dsDNA (72 base)-5′ TCG-TCG-TTT-TGT-CGT-TTT-GTC-GTT-TCG-TCG-TTT-TGT-CGT-TTT-GTC-GTT-TCG-TCG-TTT-TGT-CGT-TTT-GTC-GTT 3′

3′ AGC-AGC-AAA-ACA-GCA-AAA-CAG-CAA - AGC-AGC-AAA-ACA-GCA-AAA-CAG-CAA-AGC-AGC-AAA-ACA-GCA-AAA-CAG-CAA 5′

Figure S5. Transfer characteristics of the top-gated graphene device measured between the drain current *vs.* top gate voltage at $V_d = 0.01$ V. The measurements were made on PBS and double-stranded DNA at 10 μM concentration. The dsDNA feature is clearly shown in above.
